# Gluten-Free and Gluten-Restricted Diets in Multiple Sclerosis: A Systematic Review of Disease Activity and Patient-Reported Outcomes

**DOI:** 10.3390/nu18193190

**Published:** 2026-09-27

**Authors:** Rakan Alromayan, Reema Alshihri, Nora Aljohani, Dana Alyahya, Najeeb Qadi, Eslam Shosha

**Affiliations:** 1 College of Medicine, King Saud University, Riyadh 11461, Saudi Arabia; 2 College of Medicine, Alfaisal University, Riyadh 11461, Saudi Arabia; 3 Neuroscience Centre of Excellence, King Faisal Specialist Hospital and Research Centre, Riyadh 11211, Saudi Arabia; 4 Division of Neurology, Department of Medicine, McMaster University, Hamilton, ON L8S 4K1, Canada

**Keywords:** multiple sclerosis, gluten-free diet, gluten, wheat, elimination diet, fatigue, quality of life, systematic review

## Abstract

**Background:** Dietary modification is widely used by people with multiple sclerosis (MS), and gluten avoidance is a common self-directed strategy. Biological plausibility has been proposed through gut-barrier and immune mechanisms, but whether gluten restriction improves clinical outcomes or objective disease activity remains uncertain. **Objective:** To systematically identify, appraise, and synthesise evidence on gluten-free and gluten-restricted diets in MS, with particular attention to whether observed effects can be attributed specifically to gluten. **Methods:** The protocol was registered with PROSPERO (CRD420261459657). MEDLINE (via PubMed), Embase, Scopus, the Web of Science Core Collection, and the Cochrane Library were searched from 1 July from the inception of each database to 22 July 2026, supplemented by backward and forward citation searching. Eligible studies enrolled adults with MS and evaluated a gluten-free diet, a clearly defined gluten-restriction component, or habitual gluten exposure. Screening, extraction, and appraisal were performed independently in duplicate. RoB 2, ROBINS-I, and the Newcastle-Ottawa Scale were applied according to design, and certainty was assessed with GRADE. Clinical and methodological heterogeneity precluded meta-analysis; results were synthesised narratively following SWiM guidance. **Results:** Of 694 records identified, 392 duplicates were removed and 302 records were screened by title and abstract, of which 292 were excluded; ten reports were sought for full-text retrieval and one could not be obtained; nine full texts were assessed, three were excluded (one non-English publication and two ineligible study designs), and six studies were included. Two parallel-group controlled trials were reported as randomised, one was a randomised crossover trial, two were non-randomised interventional studies, and one was observational. Only six studies were eligible, and they were small and clinically and methodologically heterogeneous; no randomised comparison isolated gluten restriction as the sole dietary change. Clinical outcomes (fatigue, quality of life, disability) are reported separately from biological or disease-activity outcomes (relapse, magnetic resonance imaging activity, and biomarkers). Fatigue and quality of life improved most consistently in multi-component dietary trials that did not isolate gluten. Evidence specifically isolating gluten restriction was much weaker, and these results should not be read as showing that a gluten-free diet itself confers clinical benefit. The two studies reporting improved disability used adherence-based, non-randomised comparisons. By contrast, the randomised crossover trial of marked wheat/gluten reduction found no disability benefit and had a negative primary immunological endpoint. This contrast reflects substantial uncertainty and does not support a causal benefit of gluten elimination on disability. Adherence was a recurrent limitation. GRADE certainty was very low for all seven outcome domains, including fatigue and quality of life, and most of the evidence was indirect with respect to gluten elimination. **Conclusions:** In six small and heterogeneous studies, fatigue and quality of life improved mainly with multi-component dietary interventions; current evidence does not establish that gluten elimination itself confers clinical benefit, is responsible for these improvements, or modifies MS disease activity. Adequately powered randomised trials that isolate gluten elimination, objectively verify adherence, and use clinically meaningful outcomes with longer follow-up are required.

## 1. Introduction

Multiple sclerosis (MS) is a chronic immune-mediated disease of the central nervous system and a major cause of non-traumatic neurological disability in young adults [1,2]. Its clinical course is heterogeneous, most commonly beginning with a relapsing-remitting phase and, in some patients, transitioning to secondary progressive disease with disability accumulation that becomes increasingly independent of relapses [3,4].

The global prevalence and absolute burden of MS have increased over recent decades [5,6,7,8,9,10]. Contemporary estimates indicate that millions of people are affected worldwide, with substantial geographic variation in prevalence, access to diagnosis, and long-term disability burden [5,10].

MS pathobiology reflects interactions between genetic susceptibility, environmental exposures, adaptive and innate immune responses, and neurodegenerative mechanisms [2,3,11,12]. Inflammatory activity is most conspicuous early in the disease course, while axonal and neuronal injury contribute importantly to long-term disability.

Disease-modifying therapies (DMTs) substantially reduce inflammatory disease activity, particularly in relapsing MS, but they do not eliminate all symptoms or fully prevent long-term disability in every patient [13,14,15]. This has sustained interest in adjunctive strategies that may improve symptom burden, cardiometabolic health, and quality of life without replacing evidence-based DMTs [16].

A biological rationale for studying gluten restriction in MS has been proposed through the gut–brain axis. Increased intestinal permeability has been reported in MS [17,18], and experimental work supports a role for gut-derived signals in shaping neuroinflammatory immune responses [19,20]. Gliadin can trigger zonulin release and increase epithelial permeability in experimental and ex vivo systems, including non-coeliac mucosa, through pathways such as CXCR3-MyD88 signalling [21,22]. A small study has also reported altered serum zonulin in MS, although its clinical significance remains uncertain [23]. Gluten-related disorders can have extra-intestinal neurological manifestations [24,25], but these observations do not establish a gluten-driven mechanism in MS.

The gut microbiota contributes to immune regulation through microbial metabolites and interactions with regulatory and effector immune pathways. Altered microbial composition has been described in MS, and microbiota from people with MS can influence neuroinflammatory phenotypes in experimental models [26,27]. Diet is one of several determinants of microbial composition; however, direct evidence that gluten withdrawal produces MS-specific immunological benefit remains limited.

Clinical evidence is sparse. Small studies and reports have raised the possibility that gluten avoidance may influence neurological symptoms or MS-related outcomes [28,29,30], but these signals are difficult to interpret because coeliac disease and other gluten-related disorders may coexist with MS and independently respond to a gluten-free diet.

Routine gluten avoidance is not established as a disease-modifying treatment for MS. Existing studies are small and heterogeneous, with variable definitions of gluten restriction, inconsistent assessment of coeliac disease or gluten sensitivity, limited objective adherence verification, and incomplete reporting of DMT use. Outcomes range from fatigue and quality of life to Expanded Disability Status Scale (EDSS) scores, relapse activity, magnetic resonance imaging (MRI) measures, and inflammatory biomarkers.

It therefore remains unclear whether gluten elimination itself affects MS disease activity, whether any benefit is confined to symptoms or particular subgroups, and how effects vary with intervention intensity, duration, and concurrent DMT. This review systematically identifies, appraises, and synthesises the evidence on gluten-free, gluten-restricted, and related dietary exposures in MS, explicitly separating gluten-specific evidence from broader dietary interventions.

## 2. Methods

### 2.1. Protocol, Registration and Reporting

This review was conducted and is reported in accordance with the Preferred Reporting Items for Systematic Reviews and Meta-Analyses (PRISMA) 2020 statement [31]. The completed 27-item checklist is provided as Supplementary File S1. The protocol was registered with the International Prospective Register of Systematic Reviews (PROSPERO) on 24 July 2026 (CRD420261459657). A full protocol was prepared before data extraction and is held by the review team; it is available from the corresponding author on request.

The review commenced on 1 July 2026. Database searching and title-and-abstract screening had begun at the time of registration, whereas data extraction, risk-of-bias assessment, and synthesis had not. Registration therefore preceded the extraction and analysis of any outcome data but followed the start of study identification; we report this transparently rather than describe the review as fully prospectively registered. Differences between the registered protocol and the review as conducted are set out in Section 2.12.

### 2.2. Eligibility Criteria

Eligibility was defined using the PICOS (Population, Intervention, Comparator, Outcomes, and Study design) framework and is summarised in Table 1.

#### Classification by Design Tier and by Directness of the Intervention

Two characteristics of the eligible studies determine how their results may legitimately be combined, and both were recorded prospectively during extraction.

First, studies were assigned to a design tier, as registered: randomised and non-randomised interventional studies formed the primary evidence tier, and cohort, case–control, and cross-sectional studies formed the supporting tier.

Second, studies were classified by the directness with which the intervention isolated gluten. Eligible interventions proved heterogeneous in this respect: some restricted gluten alone; some restricted gluten as one element of a multi-component elimination diet that simultaneously excluded dairy, grains, legumes, and other foods; one reduced wheat and amylase-trypsin-inhibitor intake by more than 90% without achieving gluten elimination in most participants; and one measured habitual gluten intake as a continuous exposure with no dietary intervention. Combining these would conflate the effect of gluten removal with concurrent dietary change and with differences in exposure measurement. Studies were therefore labelled as follows:

- Direct: gluten restriction is the principal and essentially sole dietary change relative to the comparator.
- Indirect, multi-component: gluten restriction forms one element of a broader elimination diet.
- Indirect, partial reduction: wheat or gluten intake is reduced rather than eliminated.
- Indirect, observational exposure: no intervention is assigned; habitual gluten intake is measured and related to outcome.

Only studies classified as direct were eligible to contribute to a pooled estimate. All other categories were synthesised narratively and reported separately, so that evidence bearing specifically on gluten removal is distinguishable from evidence that does not.

### 2.3. Information Sources

We searched five bibliographic sources: MEDLINE (via PubMed), Embase (via Ovid), Scopus, the Web of Science Core Collection, and the Cochrane Library (comprising the Cochrane Database of Systematic Reviews and the Cochrane Central Register of Controlled Trials). All searches were run on a single day, 22 July 2026, from the inception of each database (earliest coverage: 1946 for MEDLINE and 1974 for Embase, and the earliest available records for Scopus, the Web of Science Core Collection, and the Cochrane Library) to the search date, with no other date restriction. The searched timeframe therefore extended from the earliest indexed records to 22 July 2026; the included studies were published between 2014 and 2025. Reference lists of all included reports were screened (backward citation searching), and articles citing each included report were identified through Scopus and the Web of Science (forward citation searching).

Grey literature was not searched, and trial and study registries were not searched separately; registry records were identified only when returned by the database searches. Both decisions depart from the review as registered and are declared in Section 2.12. The resulting potential for publication bias is considered in Section 2.11.

### 2.4. Search Strategy

The strategy combined two concept blocks with the Boolean operator AND. Block A captured the population using controlled vocabulary for multiple sclerosis and demyelinating disease, together with free-text terms for the disease and its recognised subtypes and the truncated stem demyelinat*. Block B captured the intervention and exposure using controlled vocabulary for gluten-free diet and glutens, together with free-text terms for gluten, gluten-free diet, gliadin, prolamins, wheat protein, gluten restriction, gluten exclusion, and coeliac disease. Database-specific syntax and field tags were applied throughout: MeSH and [tiab] in PubMed; exploded Emtree headings and .ti,ab,kw. in Ovid Embase; MeSH descriptors with :ti,ab,kw in CENTRAL; TS= in the Web of Science; and TITLE-ABS-KEY in Scopus. The English-language limit was applied as the final step of each search.

Coeliac disease terms were retained in Block B deliberately, because the clinical literature on gluten restriction in MS is substantially indexed under coeliac disease and gluten sensitivity; this broadens the search at the cost of a larger screening burden.

**Sensitivity of the population block.** The unqualified abbreviation “MS” was retained as a free-text term in the PubMed, Embase, and CENTRAL strategies. In isolation this term is highly non-specific, retrieving records concerning mass spectrometry, milliseconds, and unrelated abbreviations. Because Block A is combined with Block B using AND, the practical effect is limited to an increase in irrelevant records at title-and-abstract screening rather than any loss of sensitivity; it is reported here for transparency.

**Yield.** The database searches returned 277 records from Scopus, 242 from Embase, 139 from MEDLINE via PubMed, 17 from the Web of Science Core Collection, and 17 from the Cochrane Library, giving 692 records. Citation searching identified a further two reports, yielding 694 records in total.

**Records identified by citation searching.** Backward citation searching identified two reports that the database searches had not retrieved, both of which met the eligibility criteria and were included: Rodrigo et al. [32] and Wahls et al. [33]. Two of the six included studies were therefore identified only through reference-list checking. The two studies were missed for different reasons. Rodrigo et al. is published in a journal not indexed in any of the five sources searched. Wahls et al. is indexed, but the intervention block specifies gluten and wheat terms, whereas that trial characterises its intervention as a modified Paleolithic elimination diet and does not describe it in gluten terms in the title, abstract, or keywords, even though the diet excludes gluten entirely. This indicates limited sensitivity of a gluten-specific search for trials of multi-component elimination diets and is considered in Section 4.

### 2.5. Selection Process

Records were imported into Rayyan [34], and duplicates were identified and removed using its duplicate-detection function. Titles and abstracts were screened independently by two reviewers (RAs and DA) against the eligibility criteria, and the full texts of all potentially eligible records were assessed independently by two further reviewers (NA and RAr). Rayyan was used solely as a screening platform; all inclusion and exclusion decisions were made by human reviewers, and no automated relevance ranking or machine-learning prioritisation was used to include or exclude any record. Disagreements at either stage were resolved by discussion, with recourse to a third reviewer when consensus was not reached.

The flow of records from identification to inclusion is presented in Figure 1, which reports the number of records excluded at the title-and-abstract stage (n = 292), with the number excluded for each reason, and the reasons for exclusion at the full-text stage. At the title-and-abstract stage, records were excluded when they clearly did not meet the eligibility criteria in Table 1, that is, when they did not concern adults with MS, did not evaluate gluten restriction or gluten exposure, or were of an ineligible study design or publication type. A single primary reason for exclusion was recorded for each excluded record.

#### Reports Not Retrieved

One report identified by the search could not be obtained [35]. The article predates electronic publication, has no digital object identifier, and is not deposited in PubMed Central, the publisher archive, or any accessible repository; the journal ceased publication in the 1980s. Full text was sought in August 2026 through the library holdings of King Faisal Specialist Hospital and Research Centre, McGill University, and the University of Ottawa. Contacting the author was not considered feasible given the age of the report. The record is counted in Figure 1 as a report not retrieved; it did not undergo eligibility assessment and is not included among reports excluded with reasons.

### 2.6. Data Collection Process

Data were extracted into a structured spreadsheet developed for this review, comprising linked sheets for study characteristics, continuous outcomes, dichotomous outcomes, risk-of-bias judgements, a derivation log, and an issues log. The extraction form and its accompanying codebook were piloted on two studies and refined before full extraction. Extraction was performed independently in duplicate by three extractors working in pairs (RAr, RAs, and NA), with disagreements resolved by discussion. When required data were not available in a published report, the corresponding author was to be contacted.

Three procedural safeguards were applied. First, every extracted value was recorded together with the page and the specific table or figure from which it was taken, so that each entry was traceable to its source. Second, all values were independently re-entered from the source publication and compared with the first extraction; discrepancies were resolved against the original text before a value was accepted. Third, each row was colour-coded by verification status, so that values awaiting independent confirmation remained visually distinct from those verified.

Any value not read directly from the published text, whether a unit conversion or a statistic derived from other reported quantities, was recorded in the derivation log with its formula and source location. Values reported only as a threshold *p*-value, or presented only within a figure with no corresponding numeric value in the text or tables, were recorded as such. Such values were not estimated from figures and were not back-calculated, except when a report provided an exact *p*-value alongside an interpretable effect estimate, in which case the derivation was performed and logged. Outcomes not reported by a study were recorded as “not reported” and were never imputed as null or zero.

### 2.7. Data Items

For each study we extracted the first author, year of publication, country and setting, trial registration identifier, study design as described in the methods rather than the title, diagnostic criteria, eligibility criteria, number of participants allocated and analysed in each group, the intervention and comparator diets including any concomitant supplementation, the method and results of any adherence assessment, intervention duration and outcome timepoints, funding source, and declared conflicts of interest.

For each outcome we extracted the effect estimate and its dispersion as reported by the source, together with the analysis population, the timepoint, and the comparison to which any reported *p*-value referred. Dispersion measures were recorded in the form in which they were published (standard deviation, standard error, or interquartile range) and labelled accordingly, rather than converted at the point of entry.

### 2.8. Risk of Bias Assessment

Risk of bias was assessed using the instrument appropriate to each study design. Trials reported as randomised, including the crossover trial, were assessed with the Cochrane RoB 2 tool [36], applying crossover-specific guidance where relevant. Non-randomised studies of interventions were assessed with ROBINS-I [37]. The single observational study, which assigned no intervention and measured habitual dietary exposure, was assessed using the cross-sectional adaptation of the Newcastle-Ottawa Scale [38], consistent with the registered approach. Two assessors (RAr and RAs) rated each study independently and resolved disagreements by discussion; further information was to be sought from investigators when a judgement could not be reached from the published report.

The addition of ROBINS-I to the registered instruments requires explanation. Two included studies formed their comparison groups on the basis of participants’ willingness or demonstrated ability to adhere to a gluten-free diet rather than by allocation; in one, all participants were offered the diet, and those who abandoned it during the first six months were subsequently designated the control group. Allocation by adherence introduces confounding that RoB 2, which presumes randomisation, cannot represent, and that the Newcastle-Ottawa Scale, which is designed for exposure-based observational studies, does not capture. ROBINS-I contains a dedicated pre-intervention confounding domain and is the instrument recommended for this design. Its addition therefore strengthens rather than departs from the registered approach, and is declared in Section 2.12.

When a publication label conflicted with the allocation method described in the Methods, classification and risk-of-bias interpretation were based on the reported allocation process rather than the article title or abstract. This principle was applied conservatively and any discrepancy was described explicitly in the Results.

### 2.9. Effect Measures

Between-group differences in continuous outcomes are expressed as mean differences with 95% confidence intervals (CI) when studies used the same instrument, and as standardised mean differences when instruments differed. Dispersion measures were used as published. When a standard deviation was required and a report provided a standard error, the standard deviation was obtained as the standard error multiplied by the square root of the analysed sample size; when a report provided a median with interquartile range, mean and standard deviation were estimated only when the underlying distribution made this defensible. Every such conversion is recorded in the derivation log with its formula and source. Dichotomous outcomes are expressed as risk ratios or, when a study reported one, as the published odds ratio with its confidence interval. Change-from-baseline values accompanied by a paired within-participant dispersion measure were treated as paired data and were not analysed as independent group means. Within-group changes (from baseline to follow-up within a single arm) are distinguished from between-group differences throughout; comparative statements about one diet relative to another are based only on between-group comparisons, and within-group changes are labelled as such.

### 2.10. Synthesis Methods

A narrative synthesis was pre-specified as the primary approach, structured by design tier and then by outcome domain and supported by summary evidence tables, following Synthesis Without Meta-analysis (SWiM) guidance [39]. Random-effects meta-analysis with heterogeneity quantified by I^2^ was pre-specified for any outcome reported by a sufficient number of clinically and methodologically homogeneous studies.

That condition was not met for any outcome, and no meta-analysis was performed. We report the reasons in full, so that the decision is transparent. The studies comparing a strict gluten-free diet with an unrestricted diet formed their groups by adherence rather than allocation and reported different outcome metrics at markedly different follow-up durations: final values after several years in one case, and change scores at six months in the other. The trials of multi-component elimination diets do not isolate gluten, and one employed an active dietary comparator that was itself not gluten-free. One trial used a randomised crossover design contributing paired within-participant data that cannot be combined with parallel-group data without adjustment. One study was observational, measuring habitual gluten intake as a continuous score with no diet-defined groups and therefore no effect estimate to pool. A summary estimate drawn across these designs would not correspond to any answerable clinical question.

Results are accordingly presented by outcome domain within design tier and directness category, with effect estimates and confidence intervals reported as published for each study individually and no pooled estimate calculated. When studies could not be combined, we describe the direction and consistency of effects, the precision of individual estimates, and the degree to which each study’s design permits a causal interpretation.

Two multi-component dietary trials shared investigators and closely related dietary protocols [33,40]. Both involved the developer of the Wahls diet, and the WAVES publication disclosed diet-related financial interests with formal conflict-management plans. These studies were therefore interpreted as related evidence rather than fully independent replication.

### 2.11. Reporting Bias and Certainty of Evidence

Selective outcome reporting was assessed by comparing the outcomes reported in each included study with its registered protocol or trial registry entry, when one existed, and forms part of the corresponding risk-of-bias domain. Funnel plots and Egger’s test were pre-specified for any meta-analysis including at least ten studies; as no analysis approached this threshold, these methods were not applied, and publication bias is instead discussed qualitatively in light of the small and largely positive published literature and the absence of grey-literature searching.

The certainty of the body of evidence for each main outcome was assessed with the Grading of Recommendations, Assessment, Development and Evaluation (GRADE) approach [41], considering risk of bias, inconsistency, indirectness, imprecision, and publication bias. Two reviewers rated certainty independently as high, moderate, low, or very low, with disagreements resolved by discussion or a third reviewer. In line with GRADE guidance, bodies of evidence from randomised trials started at high certainty and those from non-randomised or observational studies at low certainty; each domain judged to have a serious concern was downgraded by one level and each judged very serious by two levels, so that serious concerns in three domains lowered randomised evidence from high to very low certainty. Ratings in the GRADE summary of findings (Section 3.7) refer to the body of evidence contributing to each outcome; where multi-component or partial-reduction interventions contributed, indirectness was explicitly downgraded and the narrower inference that any observed benefit was caused specifically by gluten elimination was interpreted more cautiously.

### 2.12. Deviations from the Registered Protocol

The following differences between the protocol registered on 24 July 2026 and the review as conducted are declared in accordance with PRISMA 2020.

- Addition of ROBINS-I. The registered protocol specified RoB 2 and the Newcastle-Ottawa Scale. ROBINS-I was added for non-randomised studies of interventions after screening identified two studies whose comparison groups were formed by participant adherence rather than allocation, a confounding structure that neither registered instrument rates adequately (Section 2.8).
- Addition of a directness classification. The registered protocol stratified evidence by design tier alone. A directness axis was introduced during extraction when it became apparent that eligible interventions ranged from strict gluten elimination through partial wheat reduction to observational measurement of habitual intake, and that combining them would confound gluten removal with concurrent dietary change (Section Classification by Design Tier and by Directness of the Intervention).
- Trial and study registries were not searched separately. The registered protocol lists searching trial or study registers among the methods of identifying studies, and the search-strategy document names ClinicalTrials.gov and the WHO International Clinical Trials Registry Platform. In the event, no separate registry search was conducted; registry records entered the review only when returned by the database searches. This narrows the search and is declared here rather than described as performed.
- Grey literature was not searched. The search-strategy document lists OpenGrey as a source. OpenGrey was permanently discontinued in 2021 and was not searched, and no alternative grey-literature source was substituted. The registered protocol states that only published studies would be sought, and the review as conducted is consistent with that statement.

No other deviations from the registered protocol occurred.

### 2.13. Registration, Funding and Conflicts of Interest

This review is registered with PROSPERO (CRD420261459657). It received no specific or external funding and was supported by the institutions of the review team on a non-commercial basis. No conflicts of interest were declared by any member of the review team. Financial interests disclosed by the authors of included studies are described in Section 2.10.

## 3. Results

### 3.1. Study Selection

The database searches identified 692 records, and citation searching identified a further two reports, giving 694 records in total. Of these, 392 were identified as duplicates and removed, leaving 302 unique records for title-and-abstract screening, of which 292 were excluded because they did not meet the eligibility criteria in Table 1 (ineligible study design or publication type, n = 147; ineligible population, n = 93; ineligible intervention or exposure, n = 52). Ten reports were sought for full-text retrieval; one could not be obtained [35] and is recorded as a report not retrieved. The remaining nine underwent full-text assessment, of which three were excluded (one published in a language other than English and two that did not meet the pre-specified study-design criteria). Six published studies were therefore included in the final review. The study-selection process is presented in the PRISMA flow diagram (Figure 1).

### 3.2. Study Characteristics

The six included studies were published between 2014 and 2025 and were conducted in Spain, the United States, Türkiye, Germany, and Australia. Five evaluated an assigned dietary intervention incorporating gluten or wheat restriction, whereas one evaluated habitual dietary exposure without assigning an intervention. Sample sizes ranged from 20 enrolled participants in a crossover pilot trial to 186 analysed participants in the observational study, and follow-up ranged from approximately 3 months to 5.3 years. The characteristics of the included studies are summarised in Table 2.

#### 3.2.1. Characteristics of Rodrigo et al. [32]

Rodrigo et al. [32] evaluated a gluten-free diet (GFD) compared with a regular diet in 72 individuals with relapsing-remitting multiple sclerosis (RRMS), with 36 participants in each group. Reported follow-up was 5.3 ± 1.6 years, with a median duration of 4.5 years. Although the article was titled a randomised clinical trial and the abstract described participants as being “randomly separated”, Section 2 states that all participants were initially offered a GFD and that the final groups were formed according to their subsequent adherence. Thirty-six participants who strictly maintained the GFD constituted the GFD group, while the 36 who discontinued or inconsistently followed the diet constituted the regular-diet control group. We therefore treated the study as a non-randomised, adherence-based comparative study in the risk-of-bias assessment.

All participants underwent duodenal biopsy. Eight had histologically confirmed coeliac disease, and Section 4 states that all eight were in the gluten-free diet group (22.2% versus 0%), with the authors concluding that this “has probably influenced these favourable results”. This is inconsistent with Table 2 of the same paper, which reports mild villous atrophy in 5 participants (13.9%) in the gluten-free group and 3 (8.3%) in the regular-diet group (*p* = 0.710). Baseline iron-deficiency anaemia was also more common in the gluten-free group (56% versus 22%; *p* = 0.004). Because a gluten-free diet is the definitive treatment for coeliac disease, any benefit observed in this study cannot be separated from the treatment of undiagnosed coeliac disease in part of the intervention group. The principal outcomes were annualised relapse rate (ARR), EDSS, and MRI lesion activity.

#### 3.2.2. Characteristics of Irish et al. [40]

Irish et al. [40] reported a pilot randomised controlled trial evaluating a modified Paleolithic dietary intervention (MPDI) in people with RRMS and fatigue. Thirty-four participants entered the two study arms (17 MPDI; 17 usual-diet control), but only 8 MPDI participants and 9 controls completed the intervention and contributed to the primary per-protocol analysis. The first ten assignments were made by coin flip, after which subsequent assignment was used to approximately balance baseline FSS scores between groups; this created concern about the randomisation process. The intervention lasted approximately 3 months after a 2-week run-in period.

The MPDI was broader than a gluten-free diet alone and excluded gluten, dairy products, potatoes, and legumes. Outcomes included fatigue measured with the Fatigue Severity Scale (FSS), health-related quality of life measured with the Multiple Sclerosis Quality of Life-54 (MSQOL-54), motor function measured with the 9-Hole Peg Test and walking tests, cognitive function measured with the Paced Auditory Serial Addition Test (PASAT), and exercise capacity.

#### 3.2.3. Characteristics of Wahls et al. [33]

Wahls et al. [33] conducted the WAVES randomised parallel-group clinical trial comparing a modified Paleolithic elimination diet (Wahls diet) with a low-saturated-fat diet (Swank diet) in people with RRMS. Eighty-seven participants were randomised, with 43 assigned to the Wahls diet and 44 to the Swank diet. Seventy-seven participants completed the 12-week primary endpoint and 72 completed the 24-week follow-up.

The study comprised a 12-week run-in followed by 24 weeks of intervention. Both diets were actively supported by dietary counselling, and adherence was relatively high. The primary outcomes were fatigue, assessed using the FSS and the Modified Fatigue Impact Scale (MFIS); secondary outcomes included the MSQOL-54 and the 6-Minute Walk Test.

#### 3.2.4. Characteristics of Karataş et al. [42]

Karataş et al. [42] evaluated a structured gluten-free diet in 54 female participants with RRMS, with 27 participants in the GFD group and 27 in the gluten-containing diet (GD) group at final analysis. Participants were not randomised: those willing and able to maintain a GFD were allocated to the GFD group, while those who did not commit to the diet were allocated to the GD group. Participants who failed to maintain the prescribed GFD were excluded.

The intervention lasted six months, with assessments at baseline, 3 months, and 6 months. The study assessed disability using EDSS, serum lipopolysaccharide (LPS), anthropometric measures, and dietary intake.

#### 3.2.5. Characteristics of Engel et al. [43]

Engel et al. [43] conducted a randomised, open-label, bicentric crossover pilot study evaluating a >90% wheat/gluten/α-amylase-trypsin inhibitor (ATI)-reduced diet compared with a standard wheat-containing diet. Twenty participants were initially enrolled, and 16 completed both dietary periods and contributed to the per-protocol analysis. Each dietary condition lasted 3 months, giving a total follow-up of 6 months.

The primary endpoint was an immunological measure; secondary outcomes included EDSS, ARR, serum neurofilament light chain (sNfL), and health-related quality of life measured with the 36-Item Short Form Health Survey (SF-36). Because each participant received both dietary conditions, comparisons were treated as paired crossover comparisons rather than as independent parallel-group comparisons.

#### 3.2.6. Characteristics of Temperley et al. [44]

Temperley et al. [44] investigated the association between habitual dairy and gluten intake and disease activity in MS using a retrospective cross-sectional design. Of 240 participants recruited, 54 were excluded and 186 were included in the final analysis.

Dietary dairy and gluten exposure over the preceding two years was retrospectively estimated with a study-specific dietary screener, while disease activity was assessed with NEDA-3 derived from clinical records. Quality of life was assessed with the Multiple Sclerosis International Quality of Life (MusiQoL) questionnaire. Only eight participants reported following a GFD, meaning that this study should not be interpreted as a GFD-versus-regular-diet intervention.

### 3.3. Risk of Bias

Risk of bias was assessed according to study design, using RoB 2 [36] for trials reported as randomised, ROBINS-I [37] for non-randomised intervention studies, and the cross-sectional Newcastle-Ottawa Scale adaptation [38] for the observational study.

The evidence base had several important methodological limitations. The Rodrigo and Karataş studies were not analysed as randomised trials, because allocation depended on dietary adherence or the willingness and ability to follow the GFD. This created potential for confounding and selection bias.

The Irish trial had substantial attrition, with only 17 of 34 participants completing the protocol, and its allocation process raised additional concern because only the initial assignments were explicitly random. These features reduced precision and increased risk of bias in the reported treatment effects.

The Wahls trial had substantially better retention: 77 of 87 randomised participants completed the 12-week assessment and 72 completed 24 weeks. Because dietary interventions cannot realistically be blinded to participants, subjective outcomes such as fatigue and quality of life remain potentially susceptible to performance and reporting bias.

The Engel study was randomised but open-label and had a small per-protocol sample of 16 participants. Its crossover design also requires consideration of within-participant effects and potential period and carry-over effects.

Temperley et al. was observational and retrospective, introducing potential for confounding and dietary recall and measurement bias. In particular, the dietary screener was developed for the study and was not otherwise validated.

Domain-level risk-of-bias judgements for the trials reported as randomised (RoB 2) and the non-randomised interventional studies (ROBINS-I) are presented in Figure 2, and the Newcastle-Ottawa Scale assessment of the observational study is presented in Table 3.

### 3.4. Results of Individual Studies

#### 3.4.1. Results of Rodrigo et al. [32]

At the end of follow-up, mean EDSS was 1.5 ± 1.4 in the GFD group compared with 2.1 ± 1.5 in the regular-diet group (*p* = 0.001). Mean ARR was also lower in the GFD group (0.4 ± 0.6 vs. 0.6 ± 0.6), although the authors reported this difference as not statistically significant. MRI lesion activity was observed in 10 of 36 (28%) participants in the GFD group compared with 24 of 36 (67%) in the regular-diet group (*p* = 0.001), corresponding to an odds ratio of 5.20 (95% CI 1.90 to 14.22) for MRI lesion activity in the regular-diet group compared with the reference GFD group (odds of 24/12 versus 10/26). These comparisons were based on adherence-defined groups and are therefore subject to confounding.

#### 3.4.2. Results of Irish et al. [40]

The Irish trial reported improvements in fatigue and several quality-of-life and functional outcomes among participants assigned to the MPDI. The FSS decreased by approximately 1.4 points in the MPDI group compared with an increase of approximately 0.2 points in the control group. The between-group comparison was statistically significant in the primary analysis (*p* = 0.03), while the corresponding intention-to-treat (ITT) analysis was borderline (*p* = 0.05).

Mental MSQOL improved by approximately 16.2% in the MPDI group and decreased by 1.5% in the control group (*p* = 0.02). Physical MSQOL also favoured the MPDI in the primary analysis (*p* = 0.03), although the ITT comparison was not statistically significant (*p* = 0.06).

For motor function, dominant-hand 9-Hole Peg Test completion time decreased by 15.1% in the MPDI group compared with 3.0% in controls; the ITT between-group analysis was statistically significant (*p* = 0.03). For the non-dominant hand, completion time decreased by 18.2% versus 7.4%, respectively, but the ITT comparison was not statistically significant (*p* = 0.14).

The 25-Foot Walk improved by 11.6% in the MPDI group compared with 3.0% in controls, but the ITT between-group comparison was not statistically significant (*p* = 0.20). Similarly, 6-Minute Walk distance increased by 10.7% versus 5.5%, respectively, but the between-group ITT comparison was not significant (*p* = 0.24). The reported *p* = 0.004 relates to a time effect rather than the between-group treatment effect and should therefore not be interpreted as evidence of superiority of the MPDI.

PASAT scores increased by 10.9% in the MPDI group compared with 5.5% in controls, with no statistically significant between-group ITT difference (*p* = 0.09). Overall, the findings were therefore most consistent for fatigue, mental quality of life, and dominant-hand motor performance, while evidence for walking and cognitive outcomes was less certain.

#### 3.4.3. Results of Wahls et al. [33]

Both dietary groups showed within-group reductions in fatigue at 12 weeks. FSS decreased by 0.71 ± 0.24 points in the Wahls group and 0.94 ± 0.18 points in the Swank group. MFIS decreased by 14.41 ± 2.22 points and 9.87 ± 1.93 points, respectively. These within-group changes were reported as mean ± standard error of the mean (SEM). There was no significant between-group difference in FSS change at 12 weeks.

At 24 weeks, FSS had decreased by 1.31 ± 0.29 points in the Wahls group and 1.01 ± 0.24 points in the Swank group (within-group changes), with no significant between-group difference. MFIS decreased by 19.1 ± 2.66 points in the Wahls group and 10.5 ± 2.46 points in the Swank group (within-group changes), with the 24-week between-group difference favouring the Wahls diet (*p* = 0.02).

Quality-of-life outcomes also improved within both groups. At 12 weeks, physical MSQOL-54 increased by 14.5 ± 2.63 points in the Wahls group and 6.04 ± 2.18 points in the Swank group (within-group changes), with the between-group comparison favouring Wahls (*p* ≤ 0.05). Mental MSQOL-54 increased by 11.3 ± 2.79 points in the Wahls group and 3.85 ± 2.63 points in the Swank group (within-group changes); the between-group comparison was also significant (*p* = 0.05).

At 24 weeks, physical MSQOL-54 had increased by 17.24 ± 2.84 points in the Wahls group compared with 9.25 ± 2.12 points in the Swank group (within-group changes), with the between-group comparison again favouring the Wahls diet. Mental MSQOL-54 increased by 14.0 ± 3.15 points in the Wahls group compared with 5.87 ± 2.65 points in the Swank group (within-group changes).

Neither group demonstrated a significant improvement in 6-Minute Walk distance at 12 weeks; a significant within-group improvement was reported in the Wahls group at 24 weeks.

#### 3.4.4. Results of Karataş et al. [42]

In this non-randomised comparison, the change in EDSS after six months favoured the GFD group. EDSS changed by −0.24 ± 0.61 in the GFD group compared with 0.00 ± 0.39 in the GD group (*p* = 0.042). Serum LPS also decreased in the GFD group (−377.93 ± 975.41 pg/mL) compared with an increase in the GD group (+207.00 ± 288.81 pg/mL; *p* < 0.001).

The study additionally reported significant reductions in body weight, BMI, waist circumference, hip circumference, and body-fat mass in the GFD group compared with the GD group. The authors reported that group assignment remained a significant predictor of ΔLPS, ΔEDSS, and Δwaist circumference after adjustment for age, disease duration, and change in Healthy Eating Index.

#### 3.4.5. Results of Engel et al. [43]

The pre-specified primary endpoint, a reduction in circulating pro-inflammatory T cells, was negative. Reductions in circulating non-classical monocytes and increases in classical monocytes were reported during the wheat-reduced period and were presented graphically without numeric values. The randomised crossover trial did not demonstrate a statistically significant difference in EDSS between the wheat-containing and wheat/gluten/ATI-reduced dietary periods. Mean EDSS was 2.0 ± 1.5 during the wheat-containing period and 2.3 ± 1.3 during the wheat-reduced period (*p* = 0.096). Serum neurofilament light chain was similarly unchanged (11.1 ± 7.0 vs. 11.4 ± 7.9 pg/mL; *p* = 0.187). One relapse occurred during the wheat-containing period and none during the wheat-reduced period.

A statistically significant difference was observed for the pain domain of the SF-36. Mean scores were 72.3 ± 30.4 during the wheat-containing period and 79.5 ± 25.6 during the wheat-reduced period (*p* = 0.008), indicating improved pain-related quality of life during the wheat-reduced period. Other SF-36 domains did not differ significantly.

#### 3.4.6. Results of Temperley et al. [44]

Temperley et al. reported a mean estimated dairy intake score of 37.8 ± 37.9 and a mean estimated gluten intake score of 219.6 ± 181.9 in the 186-participant cohort, expressed in the study-specific screener units.

No statistically significant association was found between estimated dairy intake and NEDA-3 status (*p* = 0.15) or between estimated gluten intake and NEDA-3 status (*p* = 0.60). There were also no significant associations between dairy intake and the individual NEDA-3 components of clinical relapse, EDSS, or MRI activity (*p* = 0.15, 0.47, and 0.11, respectively), or between gluten intake and those outcomes (*p* = 0.64, 0.22, and 0.47, respectively).

Similarly, neither estimated dairy nor estimated gluten intake was significantly associated with MusiQoL scores (*p* = 0.11 and 0.51, respectively). Multivariable analyses did not identify significant conjoint associations between dietary exposure and either NEDA-3 or MusiQoL.

### 3.5. Synthesis of Findings by Outcome

Findings are synthesised separately for clinical outcomes (fatigue, health-related quality of life, disability measured by EDSS, and motor, walking, and cognitive function; Section 3.5.1, Section 3.5.2, Section 3.5.3 and Section 3.5.4) and for biological or disease-activity outcomes (relapse, MRI activity, NEDA-3 status, and immunological or other biomarkers; Section 3.5.5, Section 3.4.4 and Section 3.4.5, and Table 4). This distinction matters because the clinical signals arose mainly from multi-component dietary interventions that did not isolate gluten, whereas the studies most specific to gluten either relied on non-randomised, adherence-defined groups or did not show a clear benefit in randomised testing. Across all outcomes, the evidence comprises only six small and clinically and methodologically heterogeneous studies, and none of the findings below should be read as showing that a gluten-free diet itself has demonstrated clinical benefit.

#### 3.5.1. Disability (EDSS)

Rodrigo et al. reported a lower final EDSS in participants adhering to a GFD (1.5 ± 1.4 vs. 2.1 ± 1.5; *p* = 0.001), while Karataş et al. reported a significantly greater reduction in EDSS over six months in the GFD group (−0.24 ± 0.61 vs. 0.00 ± 0.39; *p* = 0.042). In contrast, Engel et al. found no statistically significant difference in EDSS between the wheat-containing and wheat-reduced dietary periods (2.0 ± 1.5 vs. 2.3 ± 1.3; *p* = 0.096).

The apparent positive findings therefore came from two non-randomised, adherence-based studies, whereas the randomised crossover trial did not demonstrate a significant effect. This reduces confidence in the conclusion that gluten or wheat restriction independently improves disability. This contrast should be read as evidence of substantial uncertainty rather than as support for gluten elimination: in the two positive studies, adherence determined group membership, so differences in EDSS cannot be separated from baseline differences between participants who did and did not maintain the diet, whereas the only randomised comparison showed no disability benefit and had a negative primary immunological endpoint.

#### 3.5.2. Fatigue

Irish et al. reported a reduction in FSS of approximately 1.4 points with the MPDI compared with an increase of 0.2 points in controls, although the ITT analysis was borderline (*p* = 0.05). The WAVES trial demonstrated significant within-group reductions in FSS and MFIS in both the Wahls and Swank groups. The between-group difference in the 24-week MFIS reduction favoured the Wahls diet over the Swank diet (within-group changes of −19.1 ± 2.66 and −10.5 ± 2.46, respectively; between-group *p* = 0.02), while the corresponding between-group difference in FSS was not statistically significant. Because both arms of WAVES received a structured diet and improved, the within-group reductions cannot be attributed to any single dietary component, and the between-group comparison contrasts two multi-component diets that differ in many respects besides gluten. Likewise, the intervention in Irish et al. removed gluten together with dairy, potatoes, and legumes. These fatigue findings therefore reflect the dietary interventions tested and not gluten restriction itself.

#### 3.5.3. Health-Related Quality of Life

Irish et al. reported a 16.2% improvement in mental MSQOL in the MPDI group compared with a 1.5% decrease in controls (*p* = 0.02), while the primary-analysis physical MSQOL comparison also favoured MPDI but did not remain statistically significant in the ITT analysis (*p* = 0.06). In the WAVES trial, both diets improved physical and mental MSQOL (within-group changes), with several between-group comparisons favouring the Wahls diet. Engel et al. reported a statistically significant improvement specifically in the SF-36 pain domain during the wheat-reduced dietary period (*p* = 0.008), although the other SF-36 domains did not differ significantly. Conversely, Temperley et al. found no significant association between habitual gluten intake and MusiQoL (*p* = 0.51) or between dairy intake and MusiQoL (*p* = 0.11). These quality-of-life findings come from multi-component or partial-reduction interventions, and the only study of habitual gluten intake found no association, so they do not isolate an effect of gluten.

#### 3.5.4. Motor, Walking and Cognitive Function

In Irish et al., dominant-hand 9-Hole Peg Test performance improved more with the MPDI than with control in the ITT analysis (*p* = 0.03), while the non-dominant-hand result was not statistically significant (*p* = 0.14). Neither the 25-Foot Walk (*p* = 0.20) nor the 6-Minute Walk Test (*p* = 0.24) showed a statistically significant between-group ITT difference. PASAT also did not demonstrate a significant between-group difference (*p* = 0.09). The WAVES trial similarly found no significant change in 6-Minute Walk distance at 12 weeks in either group.

#### 3.5.5. Relapse, MRI Activity and NEDA-3

Rodrigo et al. reported fewer active MRI lesions among participants adhering to a GFD, although the allocation method limits causal interpretation, and the lower ARR in the GFD group was not statistically significant. Engel et al. recorded one relapse during the wheat-containing period and none during the wheat-reduced period, a difference that cannot be interpreted given a single event. Temperley et al. found no significant association between gluten intake and NEDA-3 status or any of its individual components.

#### 3.5.6. Safety and Tolerability

Safety data were sparse and inconsistently reported. Engel et al. was the only study to tabulate adverse events by exposure period: 11 events occurred in total, affecting 6 of 16 participants (37.5%) during the wheat-containing period and 5 of 16 (31.3%) during the wheat-reduced period (*p* = 0.709). Wahls et al. reported no serious adverse events in either arm, with similar discontinuation between groups (8 of 43 on the Wahls diet vs. 7 of 44 on the Swank diet by 24 weeks). Irish et al. reported no adverse events, although attrition was substantial and equal across arms. Karataş et al. did not report adverse events; discontinuation was asymmetric and driven by the intervention itself, with 14 of 41 participants (34%) allocated to the gluten-free diet failing to complete, 11 because of non-compliance, compared with 2 of 29 in the gluten-containing group. Rodrigo et al. reported no tolerability data. Temperley et al. was observational and did not assess harms.

No study identified a clear safety signal attributable to gluten or wheat restriction. This finding should not be over-interpreted, because adverse-event ascertainment was inconsistent and only one trial tabulated events systematically. Feasibility and adherence, rather than toxicity, emerged as the more prominent practical limitation.

#### 3.5.7. Dietary Adherence

Adherence was the dominant practical constraint, and verification methods varied widely. Only two studies used any objective biomarker to assess adherence, and only one of these used a marker specific to gluten intake. Irish et al. combined 24 h dietary recall with daily food logs and reported that gluten-containing grain intake in the intervention group fell from a mean of 4.55 ounce-equivalents at baseline to 0.00 at both one month and post-protocol, while control intake was essentially unchanged. Serum vitamin K rose by 262% in the intervention group against a 3.6% fall in controls (*p* = 0.02); however, this increase is consistent with higher intake of vitamin K-rich vegetables, a component of the diet, and is not specific to gluten avoidance. It therefore supports adherence to the dietary pattern in general but does not confirm gluten avoidance, which in this trial rests on self-reported dietary data. Engel et al. combined food questionnaires with urinary gluten immunogenic peptides: estimated intake fell by approximately 88%, from 6.6 ± 6.3 to 0.8 ± 2.4 g per day, but only 3 of 16 participants achieved a strict gluten-free diet, and gluten immunogenic peptides remained detectable in most participants during the wheat-reduced period, leading the authors to conclude that this marker is not a reliable measure of compliance when minor gluten intake is permitted.

The remaining studies relied predominantly on self-report. Karataş et al. provided individualised plans with fortnightly adherence calls, yet non-compliance accounted for 11 of 14 withdrawals from the gluten-free arm. Rodrigo et al. confirmed adherence by questioning participants and families at six-monthly visits without biochemical verification, and adherence itself determined group allocation. Temperley et al. estimated habitual intake retrospectively using a study-specific screener that was not validated. Overall, adherence was a recurrent limitation and was entangled with treatment assignment in the two studies reporting disability benefit.

### 3.6. Overall Synthesis

The six included studies provide heterogeneous evidence on gluten-free and related dietary interventions in MS. They differed substantially in design, allocation method, dietary intervention, comparator, outcome measures, and duration of follow-up. With only six studies, most of them small, and with substantial clinical and methodological heterogeneity, the evidence base is limited, and its conclusions should be interpreted cautiously; no randomised, gluten-specific comparison showed that a gluten-free diet itself yields clinical benefit.

The strongest controlled evidence supports a possible benefit of structured dietary modification for fatigue and quality of life rather than a gluten-specific effect. Irish et al. reported improvements in fatigue, mental quality of life, and dominant-hand motor function, but the trial was small, had substantial attrition, and had concerns about its allocation process. The larger WAVES trial showed clinically meaningful within-group improvements with both the Wahls and Swank diets, with selected outcomes favouring the Wahls diet in between-group comparisons. Because the Wahls intervention altered multiple dietary components and used another structured diet as the comparator, these findings cannot isolate the effect of gluten.

Evidence for disability and objective disease activity was less consistent. Rodrigo et al. and Karataş et al. reported statistically significant EDSS findings associated with a GFD, but both comparisons were non-randomised and vulnerable to adherence-related confounding. Engel et al., the randomised crossover trial that most directly tested marked wheat/gluten reduction, found no significant EDSS difference and a negative primary immunological endpoint. Temperley et al. found no association between habitual gluten intake and NEDA-3 or its components. This contrast between non-randomised positive findings and randomised and observational null findings is best regarded as evidence of substantial uncertainty rather than as support for gluten elimination.

Across the evidence base, the studies that showed the clearest fatigue and quality-of-life signals used broad multi-component diets, whereas the studies most specific to gluten either relied on non-randomised adherence-defined groups or did not show a clear benefit in randomised testing. Improvements observed across these heterogeneous designs therefore cannot be attributed specifically to gluten elimination.

The evidence was thus deemed too heterogeneous for quantitative synthesis. Differences in dietary composition, comparator conditions, study design, allocation methods, follow-up periods, outcome measures, and reporting of summary statistics precluded a clinically meaningful meta-analysis.

The relationship between intervention directness, study design, sample size, and the principal signal from each study is summarised in Figure 3.

### 3.7. Certainty of Evidence

Certainty was assessed for seven outcome domains using GRADE (Section 2.11) and is summarised in Table 4. Certainty was very low for all seven domains, both for the clinical outcomes (fatigue, quality of life, disability, and walking or functional performance) and for the biological or disease-activity outcomes (relapse or disease activity, MRI disease activity, and immunological or biomarker outcomes). For fatigue, quality of life, and walking or functional performance, the evidence came from randomised trials and therefore started at high certainty; it was downgraded by one level each for risk of bias, indirectness, and imprecision, which corresponds to very low certainty. The remaining outcomes were downgraded for the domains listed in Table 4, including inconsistency for some objective outcomes. Because the fatigue and quality-of-life evidence came mainly from multi-component diets, the evidence is even less direct for the question of whether gluten elimination itself caused those benefits. No outcome achieved moderate or high certainty.

**Table 4:** GRADE summary of findings.

GRADE Rationale	GRADE Certainty of Contributing Evidence	Summary of Findings	Studies/Participants	Outcome
Downgraded for risk of bias because of substantial attrition in Irish et al.; for indirectness because neither trial isolated gluten elimination; and for imprecision because the Irish trial was small. Randomised trials start at high certainty; three one-level downgrades give very low certainty.	⊕◯◯◯ Very low	Both randomised studies reported improvements in fatigue following dietary intervention. Irish et al. reported an improvement in FSS with the modified Paleolithic diet, while Wahls et al. reported clinically meaningful reductions in FSS and MFIS in both the Wahls and Swank groups (within-group changes). In WAVES, the between-group difference in FSS change was not significant, whereas the 24-week between-group difference in MFIS favoured the Wahls diet.	2 RCTs; 121 randomised	Fatigue
Downgraded for risk of bias, indirectness of the interventions, and imprecision. Three one-level downgrades from a high starting point (randomised trials) give very low certainty.	⊕◯◯◯ Very low	Improvements in MS-specific quality of life were reported in Irish et al. and Wahls et al.; Engel et al. reported improvement specifically in the SF-36 pain domain but not in most other domains.	3 studies; approximately 137 participants	Quality of life
Downgraded substantially for risk of bias in the non-randomised studies, inconsistency between studies, and imprecision. Indirectness also exists because Engel et al. evaluated wheat/ATI reduction rather than a conventional GFD.	⊕◯◯◯ Very low	Rodrigo et al. reported lower EDSS following GFD; Karataş et al. reported a small improvement in ΔEDSS; Engel et al. found no significant difference between wheat-reduced and wheat-containing periods.	3 studies; approximately 142 participants	Disability (EDSS)
		Rodrigo et al. reported a lower ARR that was not statistically significant; Engel et al. reported one relapse during the wheat-containing period and none during the wheat-reduced period; Temperley et al. found no significant association between gluten intake and NEDA-3 status.	3 studies; approximately 274 participants	Relapse rate/disease activity
		Rodrigo et al. reported MRI lesion activity in 28% of the GFD group versus 67% of the regular-diet group (OR 5.20, 95% CI 1.90 to 14.22, for the regular-diet group relative to the GFD reference group). Temperley et al. found no significant association between estimated gluten intake and MRI activity.	2 studies; approximately 258 participants	MRI disease activity
		Karataş et al. reported a significant reduction in serum LPS with GFD (ΔLPS −377.93 ± 975.41 vs. +207.00 ± 288.81 pg/mL; *p* < 0.001). Engel et al. found no reduction in the primary pro-inflammatory T-cell endpoint but observed changes in selected monocyte populations.	2 studies; approximately 70 participants	Immunological/biomarker outcomes
		Irish et al. reported improvement in selected functional measures, including the 9-Hole Peg Test. In WAVES, neither diet significantly improved 6-Minute Walk distance at 12 weeks; a significant within-group improvement was observed in the Wahls group at 24 weeks.	2 RCTs; 121 randomised	Walking/functional performance

GRADE certainty is shown with symbols ranging from ⊕⊕⊕⊕ (high) to ⊕◯◯◯ (very low); ⊕◯◯◯ indicates very low certainty. Participant numbers are the numbers randomised or analysed in the contributing studies (Rodrigo 72; Irish 34; Wahls 87; Karataş 54; Engel 16 completing both periods; Temperley 186). GRADE ratings describe certainty for the contributing dietary evidence at each outcome; where interventions were multi-component or only partially reduced gluten, the evidence is even less direct for the question of whether the observed effect was caused specifically by gluten elimination. Randomised evidence started at high certainty and each serious concern lowered certainty by one level, so serious concerns in three domains correspond to very low certainty. Fatigue, quality of life, disability, and walking or functional performance are clinical outcomes; relapse or disease activity, MRI disease activity, and immunological or biomarker outcomes are biological or disease-activity outcomes. ARR, annualised relapse rate; ATI, amylase-trypsin inhibitor; CI, confidence interval; EDSS, Expanded Disability Status Scale; FSS, Fatigue Severity Scale; GFD, gluten-free diet; LPS, lipopolysaccharide; MFIS, Modified Fatigue Impact Scale; MRI, magnetic resonance imaging; NEDA-3, no evidence of disease activity-3; OR, odds ratio; RCT, randomised controlled trial; SF-36, 36-Item Short Form Health Survey.

## 4. Discussion

### 4.1. Principal Findings

This systematic review examined gluten-free, gluten-restricted, and related dietary interventions in MS. Six studies met the inclusion criteria: two parallel-group controlled trials reported as randomised, one randomised crossover trial, two non-randomised intervention studies, and one observational study. The central finding is a consistent separation between evidence for broad dietary modification and evidence for gluten itself. Patient-reported fatigue and quality of life may improve with structured multi-component dietary interventions, but this rests on only six small and heterogeneous studies, and current evidence does not establish that gluten elimination itself improves fatigue, quality of life, disability, relapse activity, MRI disease activity, or disease progression. Clinical outcomes (fatigue, quality of life, disability) and biological or disease-activity outcomes (relapse, MRI activity, NEDA-3 status, and biomarkers) are therefore best interpreted separately.

Fatigue and quality of life were the most consistently improved outcomes. Irish et al. and the larger WAVES trial both reported favourable patient-reported outcomes, but each intervention changed multiple dietary components simultaneously. In WAVES, both the Wahls and Swank diets produced clinically meaningful within-group improvements, further limiting attribution to gluten. The pattern is therefore compatible with a benefit from structured dietary change, counselling, altered food quality, or other components of the interventions rather than a specific effect of gluten withdrawal.

Evidence for a gluten-specific effect on disability and disease activity was weaker. The positive EDSS and MRI findings in Rodrigo et al. arose from adherence-defined groups, with additional imbalance in coeliac disease and other baseline characteristics. Karataş et al. also reported a small EDSS improvement and lower serum LPS after six months, but allocation was non-randomised and non-completers were excluded. In contrast, Engel et al. used randomised crossover allocation and found no EDSS benefit or improvement in its primary immunological endpoint during marked wheat reduction, while Temperley et al. found no association between habitual gluten intake and NEDA-3. This contrast should be read as evidence of substantial uncertainty rather than as support for gluten elimination.

Taken together, these findings support further study of diet as an adjunctive component of MS care, but they do not support a causal claim that gluten elimination modifies MS disease activity. Figure 3 illustrates this evidence pattern by mapping intervention directness against study design strength.

### 4.2. Limitations of the Evidence Base

The evidence base identified in this review has several important limitations. First, the number of eligible studies was small, limiting the ability to establish consistency across independent investigations. Only six studies were included, and two of them shared investigators and closely related dietary protocols (Section 2.10), so they cannot be regarded as fully independent replication. Second, sample sizes were generally modest, and some studies were explicitly described as pilot or proof-of-concept trials.

In addition, there was substantial clinical and methodological heterogeneity. The studies differed in the type of dietary intervention, comparator, participant characteristics, follow-up duration, and outcome measures. In two studies, groups were formed according to whether participants maintained the assigned diet rather than by allocation; the resulting comparisons therefore risk confounding dietary adherence with the characteristics of participants who chose to maintain the intervention. Objective outcomes included EDSS, MRI activity, relapse rate, and biomarkers, whereas patient-reported outcomes included fatigue and quality of life. The observational study by Temperley et al. further deviated from the intervention trials in that the dietary exposure was retrospectively estimated rather than assigned. Together with the small number of studies, this heterogeneity limits the strength of any conclusion about gluten specifically, and the evidence isolating gluten restriction is considerably weaker than the evidence for multi-component dietary interventions.

Furthermore, several studies evaluated broader dietary patterns rather than isolated gluten elimination. This substantially limits the ability to answer the specific causal question of whether gluten itself affects MS outcomes. The methodological limitations identified in the risk-of-bias assessment reduce confidence in several of the positive findings. Randomised studies provide greater protection against confounding, but some were small and had appreciable attrition, while the studies reporting improvements in disability were non-randomised.

Because of this heterogeneity, a quantitative meta-analysis would have risked combining clinically and methodologically dissimilar interventions and outcomes. The decision to use a narrative synthesis was therefore appropriate and allowed the findings to be interpreted according to study design, intervention, and outcome, rather than producing a potentially misleading pooled estimate.

### 4.3. Strengths of the Evidence Base

Despite these limitations, the evidence base has several strengths. It includes controlled and randomised designs, clinically meaningful patient-reported outcomes, objective disease measures, and studies spanning direct GFD interventions, partial wheat reduction, broader elimination diets, and habitual exposure. This diversity permits an important distinction between a signal for structured dietary intervention and a gluten-specific therapeutic effect, even though it prevents quantitative pooling.

### 4.4. Limitations of the Review Process

Several limitations of the review process should also be considered. The small number of eligible studies limited the ability to investigate potential sources of heterogeneity or to conduct meaningful subgroup analyses. The absence of sufficiently comparable outcome data also precluded quantitative synthesis and limited direct comparison of effect magnitudes.

Differences in intervention definitions meant that the review necessarily incorporated studies evaluating gluten-free diets alongside broader dietary interventions that included gluten elimination. Although these were distinguished during synthesis, this heterogeneity introduces uncertainty when drawing conclusions specifically about gluten.

Two of the six included studies were identified only through reference-list checking, and one was not retrievable. The gluten-specific search strategy had limited sensitivity for trials of multi-component elimination diets that are not described in gluten terms, and grey literature and trial registries were not searched separately (Section 2.4 and Section 2.12). Relevant unpublished or non-English studies may therefore have been missed, and the small, largely positive published literature leaves the possibility of publication bias that could not be assessed formally.

### 4.5. Clinical Implications

The findings do not provide sufficient evidence to recommend a gluten-free diet as a disease-modifying treatment for MS. Patients with coeliac disease or another established gluten-related disorder should follow condition-specific dietary guidance, but routine gluten avoidance solely to modify MS activity is not supported by the current evidence. Symptomatic benefit from broader dietary improvement remains plausible, particularly for fatigue and quality of life, but should not be conflated with disease modification.

Structured dietary modification can be considered within holistic MS care when aligned with patient preferences and nutritional adequacy. Clinicians should discuss feasibility, cost, adherence, unintended weight loss or nutrient deficiency, and the importance of continuing evidence-based DMTs when indicated.

### 4.6. Future Research

Future research should prioritise adequately powered randomised controlled trials that isolate gluten elimination from other dietary changes. Trials should pre-specify a clinically credible gluten-free comparator contrast, objectively verify exposure where feasible, maintain stable DMT background therapy, and use blinded outcome assessment for objective endpoints. Coeliac disease and other gluten-related conditions should be excluded, stratified, or analysed separately so that a therapeutic response to treatment of an established gluten disorder is not misattributed to MS.

The potential contribution of other components of wheat should also be investigated. Engel et al. specifically focused on wheat reduction and ATIs rather than gluten alone, and their findings suggest that the biological effects of wheat restriction may not be attributable exclusively to gluten. Future studies could therefore compare strict gluten-free diets with wheat-reduced or ATI-reduced interventions to determine whether different components of wheat have independent effects.

Finally, future studies should report complete numerical data, including between-group effect estimates, confidence intervals, denominators at each assessment point, protocol deviations, adherence data, and adverse events. Harmonised outcomes and longer follow-up would improve comparability and could make a future quantitative synthesis clinically meaningful.

## 5. Conclusions

This systematic review, based on only six small and heterogeneous studies, found a signal that multi-component dietary modification may improve fatigue and quality of life in people with MS (very low certainty), but the available evidence does not establish that gluten elimination is the active component or that a gluten-free diet itself confers clinical benefit. Evidence for disability, relapse activity, MRI disease activity, and biomarkers was inconsistent and of very low certainty, with the positive disability findings arising from non-randomised, adherence-defined comparisons and contrasting with the null result of the randomised crossover trial; this reflects substantial uncertainty rather than support for gluten elimination. The current evidence therefore does not support a gluten-free diet as a disease-modifying treatment for MS.

Adequately powered trials that specifically isolate gluten elimination are required, with rigorous adherence assessment, careful handling of coeliac disease and gluten sensitivity, clinically meaningful outcomes, and longer follow-up. Until such evidence is available, gluten avoidance should be regarded as an investigational adjunct rather than an established MS therapy.

## Figures and Tables

**Figure 1:**
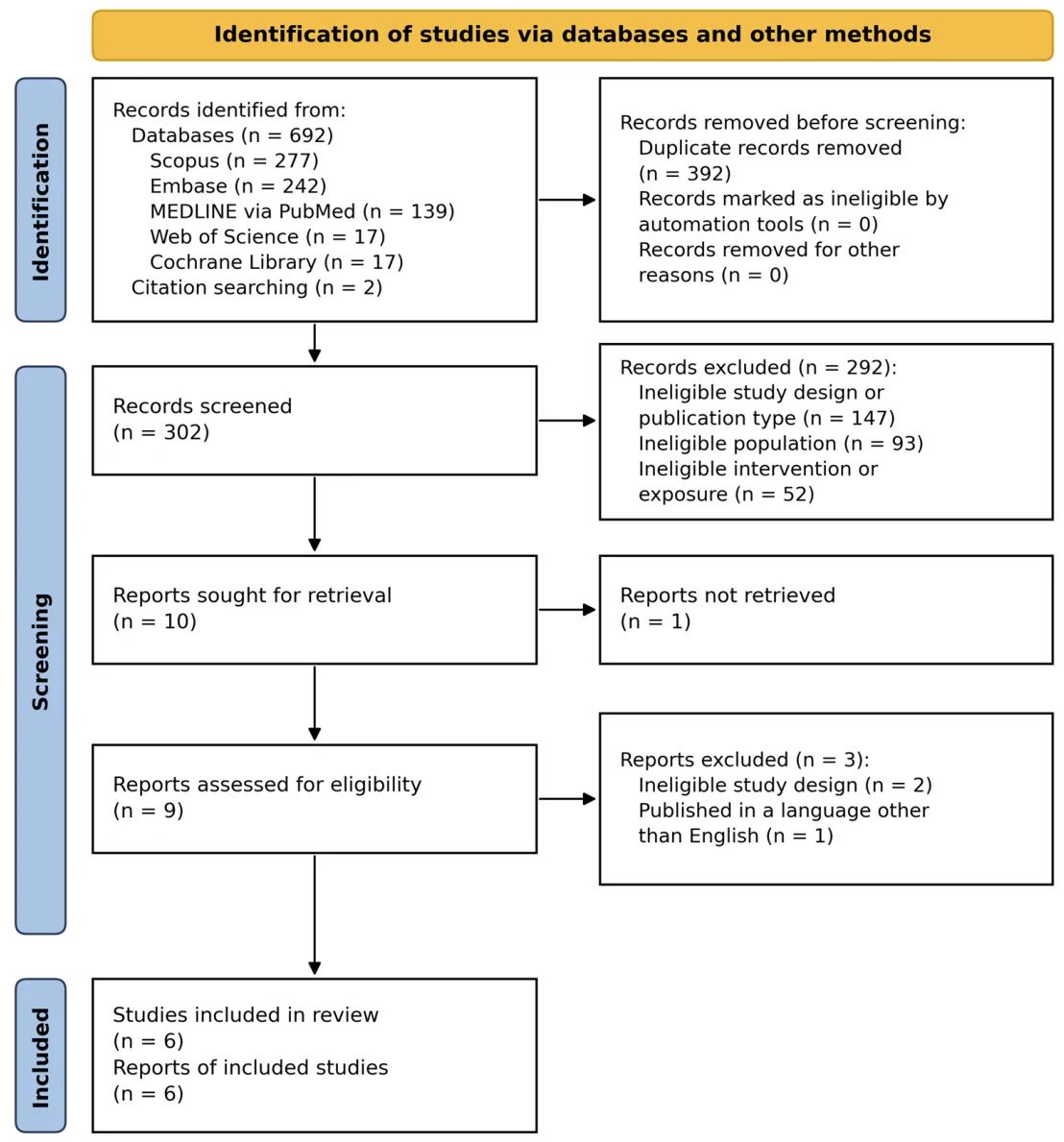
PRISMA 2020 flow diagram of study identification, screening, retrieval, eligibility assessment, and inclusion. Records excluded at the title-and-abstract stage (n = 292) did not meet the eligibility criteria in Table 1 (ineligible study design or publication type, n = 147; ineligible population, n = 93; ineligible intervention or exposure, n = 52). Reasons for exclusion at the full-text stage were publication in a language other than English (n = 1) and ineligible study design (n = 2); one report could not be retrieved and did not undergo eligibility assessment (n = 1).

**Figure 2:**
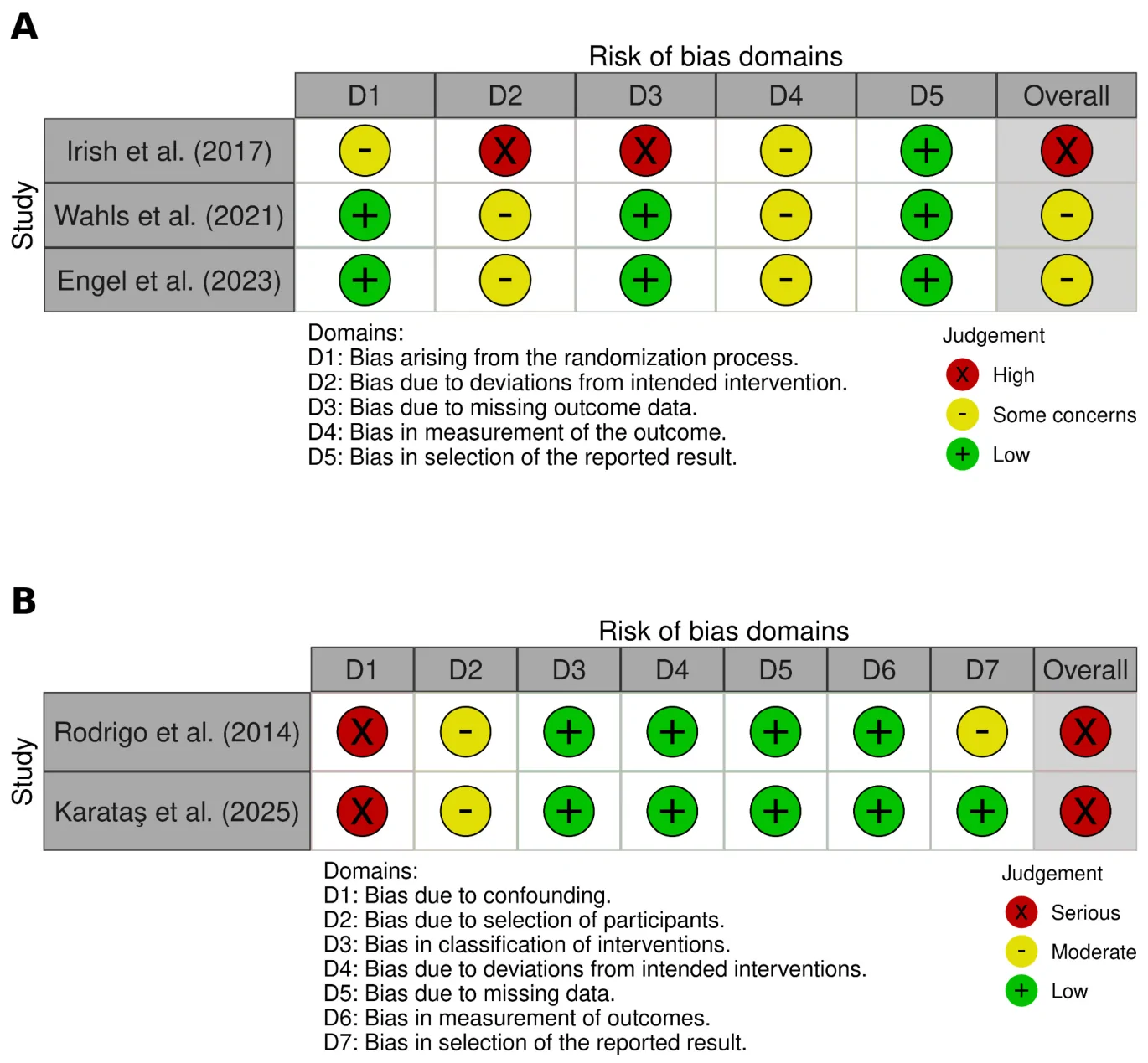
Risk−of−bias assessment of the interventional studies. (**A**) RoB 2 judgements for trials reported as randomised by Irish et al. [40], Wahls et al. [33], and Engel et al. [43]. (**B**) ROBINS-I judgements for the non-randomised adherence-based studies by Rodrigo et al. [32] and Karataş et al. [42].

**Figure 3:**
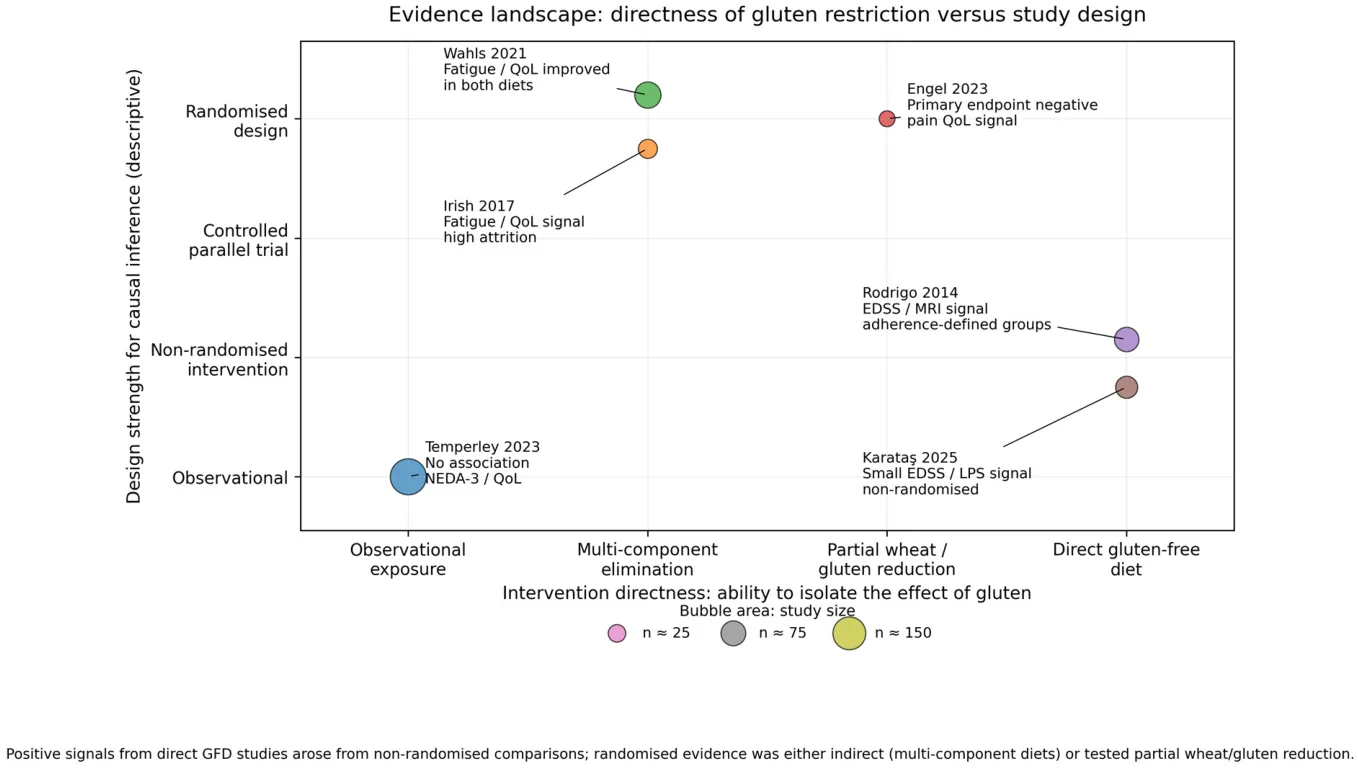
Evidence landscape by intervention directness and study design. Horizontal position reflects how specifically the intervention isolated gluten; vertical position reflects design class for causal inference, and bubble area is proportional to study size. Labels summarise the principal signal from each study. The figure is descriptive and is not an effect-size meta-analysis. The studies shown are Rodrigo et al. [32], Irish et al. [40], Wahls et al. [33], Karataş et al. [42], Engel et al. [43], and Temperley et al. [44].

**Table 1:** Eligibility criteria.

Excluded	Included	Parameter
Cohorts restricted to participants whose primary diagnosis was coeliac disease or inflammatory bowel disease; paediatric-only populations.	Adults aged 18 years or older with multiple sclerosis diagnosed by recognised criteria (e.g., McDonald), of any subtype: relapsing-remitting, secondary progressive, primary progressive, or clinically isolated syndrome. Eligible irrespective of concomitant disease-modifying therapy.	Population
Studies without a clearly defined gluten-restriction component; studies of nutritional supplements alone, with no dietary pattern.	A gluten-free diet, or any dietary pattern incorporating a clearly defined gluten-restriction component.	Intervention
None specified.	A gluten-containing or unrestricted diet, usual care, or another defined dietary intervention. Single-arm studies were eligible and reported descriptively where they contributed disease-activity data.	Comparator
Studies reporting none of the listed outcomes in extractable form.	At least one of: annualised relapse rate or relapse occurrence; disability progression (EDSS); MRI lesion activity; fatigue; health-related quality of life; inflammatory biomarkers; gut microbiome composition or intestinal permeability; no evidence of disease activity-3 (NEDA-3) status; dietary adherence; adverse effects.	Outcomes
Single case reports and small case series; narrative and systematic reviews; editorials and letters; conference abstracts without extractable data. Preclinical studies were reserved for a separate mechanistic narrative.	Primary evidence: randomised controlled trials and non-randomised interventional studies. Supporting evidence: prospective and retrospective cohort, case–control, and cross-sectional studies.	Study design
Non-English publications.	Any country and any care setting. English-language publications, from database inception to 22 July 2026 (see Section 2.3).	Setting, language, dates

**Table 2:** Characteristics of included studies.

Main Outcomes Assessed	Follow-Up	Comparator	Intervention/Exposure	Population	Sample Size	Study Design	Country	Study
EDSS; ARR; MRI disease activity	5.3 ± 1.6 years (median 4.5 years)	Regular diet	Gluten-free diet	Adults with RRMS	72 (36 GFD; 36 regular diet)	Prospective comparative study; allocation based on adherence	Spain	Rodrigo et al. (2014) [32]
FSS; MSQOL-54; 9-Hole Peg Test; 25-Foot Walk; 6-Minute Walk; PASAT	~3 months following 2-week run-in	Usual diet	Modified Paleolithic dietary intervention (MPDI), including elimination of gluten, dairy, potatoes, and legumes	Adults with RRMS and fatigue	34 randomised (17 MPDI; 17 control); 17 completed	Pilot controlled trial reported as randomised; allocation process raised concerns	USA	Irish et al. (2017) [40]
FSS; MFIS; MSQOL-54; 6-Minute Walk Test	24 weeks following 12-week run-in	Low-saturated-fat Swank diet	Modified Paleolithic elimination diet (Wahls diet)	Adults with RRMS and moderate-to-severe fatigue	87 randomised (43 Wahls; 44 Swank)	Randomised parallel-group controlled trial (WAVES)	USA	Wahls et al. (2021) [33]
EDSS; serum LPS; anthropometric measures; dietary intake	6 months	Gluten-containing diet	Gluten-free diet	Women with RRMS	54 (27 GFD; 27 GD)	Non-randomised controlled intervention study	Türkiye	Karataş et al. (2025) [42]
Immunological outcomes; EDSS; ARR; sNfL; SF-36; anthropometric measures	3 months per dietary period; 6 months total	Standard wheat-containing diet	>90% wheat/gluten/ATI-reduced diet	Adults with MS	20 enrolled; 16 completed both periods	Randomised, open-label, crossover pilot trial	Germany	Engel et al. (2023) [43]
NEDA-3; relapse; EDSS; MRI activity; MusiQoL	Retrospective dietary exposure over previous 2 years	Higher vs. lower habitual intake	Habitual gluten and dairy intake	Adults with MS, including different MS phenotypes	186 analysed	Retrospective cross-sectional observational study	Australia	Temperley et al. (2023) [44]

ARR, annualised relapse rate; ATI, amylase-trypsin inhibitor; EDSS, Expanded Disability Status Scale; FSS, Fatigue Severity Scale; GD, gluten-containing diet; GFD, gluten-free diet; LPS, lipopolysaccharide; MFIS, Modified Fatigue Impact Scale; MPDI, modified Paleolithic dietary intervention; MRI, magnetic resonance imaging; MSQOL-54, Multiple Sclerosis Quality of Life-54; MusiQoL, Multiple Sclerosis International Quality of Life questionnaire; NEDA-3, no evidence of disease activity-3; PASAT, Paced Auditory Serial Addition Test; RRMS, relapsing-remitting multiple sclerosis; SF-36, 36-Item Short Form Health Survey; sNfL, serum neurofilament light chain.

**Table 3:** Newcastle-Ottawa Scale assessment of the observational study.

Overall	Outcome Assessment	Comparability/Confounding	Exposure Ascertainment	Non-Response/Exclusions	Sample Size	Sample Representativeness	Study
6/7	★	★★	–	★	★	★	Temperley et al. (2023) [44]

Stars indicate criteria met within each domain (maximum 7 for the cross-sectional adaptation of the scale). No star was awarded for exposure ascertainment because the dietary screener was study-specific and not validated.

## Data Availability

No new data were created or analyzed in this study. Data sharing is not applicable to this article. The data-charting extraction spreadsheet will be made available via the Open Science Framework (OSF) upon publication.

## Supplementary Materials

The following supporting information can be downloaded at: https://www.mdpi.com/article/10.3390/nu18193190/s1, Supplementary File S1: PRISMA 2020 checklist.
