# Supplementary material for: Gluten-Free and Gluten-Restricted Diets in Multiple Sclerosis: A Systematic Review of Disease Activity and Patient-Reported Outcomes
Source: Nutrients. 2026 Sep 27;18(19):3190. doi: 10.3390/nu18193190 (PMC13667108; doi:10.3390/nu18193190)
Supplement: Supplementary file 1 [file nutrients-18-03190-s001.zip › nutrients-4548662-supplementary.pdf]

Supplementary File S1. PRISMA 2020 Checklist

Gluten-Free and Gluten-Restricted Diets in Multiple Sclerosis: A Systematic Review of Disease Activity and Patient-Reported Outcomes

Section and Topic	Item #	Checklist item	Location where item is reported
**TITLE**			
Title	1	Identify the report as a systematic review.	Title page
**ABSTRACT**			
Abstract	2	See the PRISMA 2020 for Abstracts checklist.	Abstract
**INTRODUCTION**			
Rationale	3	Describe the rationale for the review in the context of existing knowledge.	Section 1 (Introduction), paragraphs 1–6
Objectives	4	Provide an explicit statement of the objective(s) or question(s) the review addresses.	Section 1, final paragraph
**METHODS**			
Eligibility criteria	5	Specify the inclusion and exclusion criteria for the review and how studies were grouped for the syntheses.	Section 2.2, Table 1; Section 2.2.1
Information sources	6	Specify all databases, registers, websites, organisations, reference lists and other sources searched or consulted to identify studies. Specify the date when each source was last searched or consulted.	Section 2.3
Search strategy	7	Present the full search strategies for all databases, registers and websites, including any filters and limits used.	Section 2.4; Supplementary Table S1
Selection process	8	Specify the methods used to decide whether a study met the inclusion criteria of the review, including how many reviewers screened each record and each report retrieved, whether they worked independently, and if applicable, details of automation tools used in the process.	Section 2.5, 2.5.1
Data collection process	9	Specify the methods used to collect data from reports, including how many reviewers collected data from each report, whether they worked independently, any processes for obtaining or confirming data from study investigators, and if applicable, details of automation tools used in the process.	Section 2.6
Data items	10a	List and define all outcomes for which data were sought. Specify whether all results that were compatible with each outcome domain in each study were sought, and if not, the methods used to decide which results to collect.	Section 2.2, Table 1 (Outcomes); Section 2.7
Data items	10b	List and define all other variables for which data were sought. Describe any assumptions made about any missing or unclear information.	Section 2.7
Study risk of bias assessment	11	Specify the methods used to assess risk of bias in the included studies, including details of the tool(s) used, how many reviewers assessed each study and whether they worked independently, and if applicable, details of automation tools used in the process.	Section 2.8
Effect measures	12	Specify for each outcome the effect measure(s) used in the synthesis or presentation of results.	Section 2.9
Synthesis methods	13a	Describe the processes used to decide which studies were eligible for each synthesis.	Section 2.2.1; Section 2.10
Synthesis methods	13b	Describe any methods required to prepare the data for presentation or synthesis, such as handling of missing summary statistics, or data conversions.	Section 2.6, 2.7 (derivation log); Section 2.9
Synthesis methods	13c	Describe any methods used to tabulate or visually display results of individual studies and syntheses.	Section 2.10; Table 2, Table 4; Figures 2 and 3
Synthesis methods	13d	Describe any methods used to synthesize results and provide a rationale for the choice(s). If meta-analysis was performed, describe the model(s) and method(s) used.	Section 2.10 (narrative synthesis per SWiM guidance; pre-specified meta-analysis criteria not met — no pooled analysis performed)

Synthesis methods	13e	Describe any methods used to explore possible causes of heterogeneity among study results.	Section 2.2.1 (directness classification); Sections 3.5–3.6
Synthesis methods	13f	Describe any sensitivity analyses conducted to assess robustness of the synthesized results.	Not applicable — no meta-analysis was performed (Section 2.10)
Reporting bias assessment	14	Describe any methods used to assess risk of bias due to missing results in a synthesis.	Section 2.11
Certainty assessment	15	Describe any methods used to assess certainty (or confidence) in the body of evidence for an outcome.	Section 2.11
**RESULTS**			
Study selection	16a	Describe the results of the search and selection process, from the number of records identified to the number of studies included, ideally using a flow diagram.	Section 3.1; Figure 1
Study selection	16b	Cite studies that might appear to meet the inclusion criteria, but which were excluded, and explain why.	Section 3.1; Section 2.5.1 (report not retrieved)
Study characteristics	17	Cite each included study and present its characteristics.	Section 3.2, 3.2.1–3.2.6; Table 2
Risk of bias in studies	18	Present assessments of risk of bias for each included study.	Section 3.3; Figure 2; Table 3
Results of individual studies	19	For all outcomes, present summary statistics for each group and an effect estimate and its precision for each study.	Section 3.4, 3.4.1–3.4.6
Results of syntheses	20a	Briefly summarize the characteristics and risk of bias among studies contributing to the synthesis.	Section 3.5, 3.6
Results of syntheses	20b	Present results of all statistical syntheses conducted. If meta-analysis was done, present summary estimates and heterogeneity measures.	Not applicable — no meta-analysis performed; results presented narratively (Section 3.5–3.6)
Results of syntheses	20c	Present results of all investigations of possible causes of heterogeneity among study results.	Section 3.5–3.6; Figure 3 (directness vs. design)
Results of syntheses	20d	Present results of all sensitivity analyses conducted to assess the robustness of the synthesized results.	Not applicable — no meta-analysis performed
Reporting biases	21	Present assessments of risk of bias due to missing results for each synthesis assessed.	Section 2.11; Section 4.4
Certainty of evidence	22	Present assessments of certainty in the body of evidence for each outcome assessed.	Section 3.7; Table 4
**DISCUSSION**			
Discussion	23a	Provide a general interpretation of the results in the context of other evidence.	Section 4.1
Discussion	23b	Discuss any limitations of the evidence included in the review.	Section 4.2
Discussion	23c	Discuss any limitations of the review processes used.	Section 4.4
Discussion	23d	Discuss implications of the results for practice, policy, and future research.	Section 4.5, 4.6
**OTHER INFORMATION**			
Registration and protocol	24a	Provide registration information for the review, including register name and registration number, or state that the review was not registered.	Section 2.1; Section 2.13 (PROSPERO CRD420261459657)
Registration and protocol	24b	Indicate where the review protocol can be accessed, or state that a protocol was not prepared.	Section 2.1 (available from corresponding author on request)
Registration and protocol	24c	Describe and explain any amendments to information provided at registration or in the protocol.	Section 2.12
Support	25	Describe sources of financial or non-financial support for the review, and the role of the funders or sponsors.	Section 2.13; Declarations (Funding)
Competing interests	26	Declare any competing interests of review authors.	Section 2.13; Declarations (Conflicts of Interest)
Availability of data, code and other materials	27	Report which of the following are publicly available and where they can be found: template data collection forms; data extracted from included studies; data used for all analyses; analytic code; any other materials used in the review.	Declarations (Data Availability)

From: Page MJ, McKenzie JE, Bossuyt PM, et al. The PRISMA 2020 statement: an updated guideline for reporting systematic reviews. BMJ 2021;372:n71. doi:10.1136/bmj.n71. For more information, visit: http://www.prisma-statement.org/
